# P-289. Assessing Knowledge of HIV Pre-exposure Prophylaxis before and after Resident Education

**DOI:** 10.1093/ofid/ofaf695.510

**Published:** 2026-01-11

**Authors:** Andrea Grimbergen, Taylor Yakubik, Ian Fladie, Lauren Sisco

**Affiliations:** Baylor Scott & White - Temple, Temple, TX; Baylor Scott and White, Temple, Texas; Baylor Scott and White, Temple, Texas; Baylor Scott & White Health - Temple, Temple, Texas

## Abstract

**Background:**

Pre-exposure prophylaxis (PrEP) is highly effective at preventing human immunodeficiency virus (HIV) infection and reduces risk of transmission up to 99% when taken optimally.^1^ However, only 30% of eligible patients in U.S. received PrEP in 2020.^2^ While over 90% of primary care physicians (PCPs) have an awareness of PrEP, less than half have prescribed or referred out for PrEP.^7-9^ Lack of knowledge with managing PrEP has been noted as a major barrier to prescription for PCPs specifically.^4, 5^ This may result in fewer prescriptions for eligible patients, especially amongst trainees. This quality improvement (QI) project aimed to assess internal medicine resident PrEP prescribing competency and comfort before and after educational intervention designed to facilitate appropriate PrEP prescription in accordance with USPSTF guidelines.

**Methods:**

Second- and third-year internal medicine residents completed an hour-long didactic course reviewing the purpose, mechanism of action, side effects and eligibility criteria of PrEP. Residents completed pre-, post- (1-month), and follow-up (6-month) intervention surveys to characterize prescribing competency and comfort using Likert scales. Out of a residency cohort of 45 trainees, 25 completed the pre-intervention survey, and 15 completed the post-intervention survey. Data was analyzed using descriptive statistics and Wilcoxon’s Signed-Rank test.

**Results:**

Following our intervention, residents felt significantly more confident in their knowledge of PrEP mechanism of action (p=0.001), side effect profile (p = 0.001) and eligibility criteria (p =0.002), as well as significantly more comfortable prescribing PrEP (p=0.007). The most common barrier cited to PrEP prescription was lack of knowledge with additional concerns regarding possible cost to the patient or lack of preceptor support.

**Conclusion:**

While this QI project was limited by small sample size, it emphasizes how brief educational interventions may have significant impact on trainee knowledge and comfort with PrEP prescription. Future QI projects may examine knowledge retention over time, as well as PrEP prescription patterns by examining proportion of PrEP-eligible patients with active PrEP prescriptions in resident clinic.

**Disclosures:**

All Authors: No reported disclosures

